# Relationships Between Living in Ethnic Enclaves and Cancer Outcomes: A Systematic Review of Literature

**DOI:** 10.1007/s40615-025-02439-0

**Published:** 2025-04-21

**Authors:** Han Shen, Robert O. Vos, Lihua Liu

**Affiliations:** 1 Spatial Sciences Institute, Dornsife College of Letters, Arts and Sciences, University of Southern California, Los Angeles, CA USA; 2 Department of Population and Public Health Sciences, Keck School of Medicine, University of Southern California, Los Angeles, CA USA; 3 Los Angeles Cancer Surveillance Program, Los Angeles, CA USA

**Keywords:** Ethnic enclaves, Cancer outcomes, Health and place, Racial and ethnic health disparities, Social determinants of health

## Abstract

**Background:**

Ethnic enclaves (EEs) are culturally distinct neighborhoods, and complex relationships may exist between living in EEs and cancer outcomes. Given the increasing demographic diversity, expansion of EEs, and ongoing burden of cancer in the U.S., a growing body of literature focuses on the enclave-cancer relationships. This review aims to describe the scope, assess methods, synthesize existing evidence, and identify research gaps on this topic.

**Methods:**

Following the Preferred Reporting Items for Systematic Reviews and Meta-Analyses (PRISMA) guidelines, we found 34 empirical studies, published between January 2000 and May 2023, that examined how living in EEs may relate to various cancer outcomes, including incidence, diagnosis, treatment, and survival.

**Results:**

Most studies examined the relationships between residing in Hispanic or Asian enclaves and cancer incidence or survival in California. Studies used divergent methods to identify EEs, and results were mixed among null, protective, and risk findings. Studies explored several cancer types, with breast and colon cancers being the most studied, and findings also varied by patients' race and ethnicity, sex, and neighborhood socioeconomic status (SES). The small number of studies limits the power to draw statistical conclusions from the existing literature.

**Conclusions:**

More research is needed to adequately explore enclave-cancer relationships among diverse populations across different U.S. regions, and to comprehensively investigate potential moderators and mediators connecting EEs and cancer outcomes. Future studies may benefit from conceptualizing and operationalizing EEs based on demographic, geographic, economic, and cultural characteristics, and recognizing varying SES, immigrant concentrations, and racial/ethnic subgroup compositions across EEs.

**Supplementary Information:**

The online version contains supplementary material available at 10.1007/s40615-025-02439-0.

## Introduction

Cancer is the second leading cause of death in the U.S. and racial and ethnic disparities in cancer outcomes persist [1]. Compared to non-Hispanic Whites, racial and ethnic minorities and immigrants often have lower cancer screening rates, later diagnosis, and higher mortality [1, 2]. Cancer risks and outcomes are influenced by various biologic, social, and environmental factors [3]. Neighborhood environment, as one of the key social determinants of health, plays critical roles in residents’ cancer outcomes through different pathways, such as health behaviors, healthcare access, social support, employment opportunities, and exposures to environmental hazards [4, 5]. Ethnic enclaves (EEs) are culturally and ethnically distinct areas that feature high concentrations of a specific racial/ethnic group population and/or ethnic-specific businesses and organizations; they are often destinations for new immigrants as well as preferred residential areas for later generations [6, 7]. These areas allow new immigrants to quickly get employment, collect information on the host society, and accumulate social capital [8]. Since 1965, immigrant populations in the U.S. have been rapidly increasing, and immigrants’ sociodemographic characteristics have shifted [9]. EEs have further expanded and developed into different forms (e.g., immigrant enclaves and ethnoburbs), housing many racial and ethnic minorities [7, 10, 11].

EEs may have complex effects on residents’ cancer outcomes across the continuum, including incidence, diagnosis, treatment, and survival. Long-term exposures to the social and built environment of EEs may influence health behaviors and in turn be associated with cancer etiology and survival outcomes [5]. The EE effects on health beliefs and healthcare access may affect cancer screening uptake and cancer treatment quality. For example, EE residents may experience less discrimination and receive strong social support from co-ethnic neighbors, friends, and relatives, leading to lower levels of stress and reduced barriers to healthcare [6, 12]. EEs may also provide ethnic-specific services, including traditional food options and culturally competent health providers, promoting healthy diets and health care utilization [13]. However, EE residents are likely to preserve traditional cultural values, including some negative healthcare beliefs (e.g., cancer stigma and fatalism), which may be related to a reluctance to screen for cancer and delayed cancer treatments [14]. Furthermore, EEs differ in neighborhood socioeconomic status (SES); low SES EEs may exert detrimental influences on residents’ health due to limited access to quality care, high exposure to environmental risks, unhealthy food environment, or low neighborhood walkability [13, 15]. Understanding the relationships between living in EEs and cancer risks could guide the development of public health interventions targeting high-risk populations and communities; it has important implications for meeting health needs and reducing health disparities among racial and ethnic minorities.

A growing number of studies have examined the relationships between EEs and various cancer outcomes among different populations, benefiting from the availability of population-based cancer registry data. While previous reviews have discussed the relationships between racial segregation or, more broadly, ethnic density and cancer outcomes [16, 17], this review provides new insights by focusing on cancer studies that examined EEs, a related yet different concept from racial segregation and ethnic density. Many racial segregation measures capture the global residential patterns across the area and are often applied to segregations between White and Black populations, whereas EEs are local clusters of concentrated racial/ethnic populations [18, 19]. In addition to the high local ethnic density, EEs also feature clustering of ethnic-specific businesses and organizations. The specific social and built environment of EEs may have combined impacts on residents’ cancer outcomes. By reviewing most recent cancer studies that self-identify as adopting the concept of EE and employ a related measure, this review focuses on the conceptualization and operationalization of EE in cancer studies and aims to synthesize findings on enclave-cancer relationships, discuss methodological challenges, and identify potential research gaps in current literature. This review may inform future studies on under-researched populations, regions, cancer types and outcomes, help cancer researchers better delineate EEs, and facilitate further examination of potential pathways linking EEs and cancer outcomes.

## Methods

This systematic review followed the Preferred Reporting Items for Systematic Reviews and Meta-Analyses (PRISMA) checklist [20]. To identify studies that examined the enclave-cancer associations, search terms were determined as a list of the following keywords combined with Boolean operators: ((ethnic enclave) OR (immigrant enclave) OR (ethnic density) OR (residential segregation) OR (racial segregation) OR (neighborhood)) AND ((immigrant) OR (migrant) OR (foreign-born)) AND (cancer). The search terms were applied to five databases, including PubMed, Embase, PsycINFO, Sociological Abstracts, and Web of Science. The search was conducted in June 2023 and limited results to studies that were written in English. As the immigration patterns, demographic compositions, neighborhood environments, and healthcare resources evolve over time, potentially influencing EE features and cancer outcomes, this review focuses on more recent studies published between January 2000 and May 2023 to ensure relevance to contemporary health disparities and cancer control efforts.

**Fig. 1:**
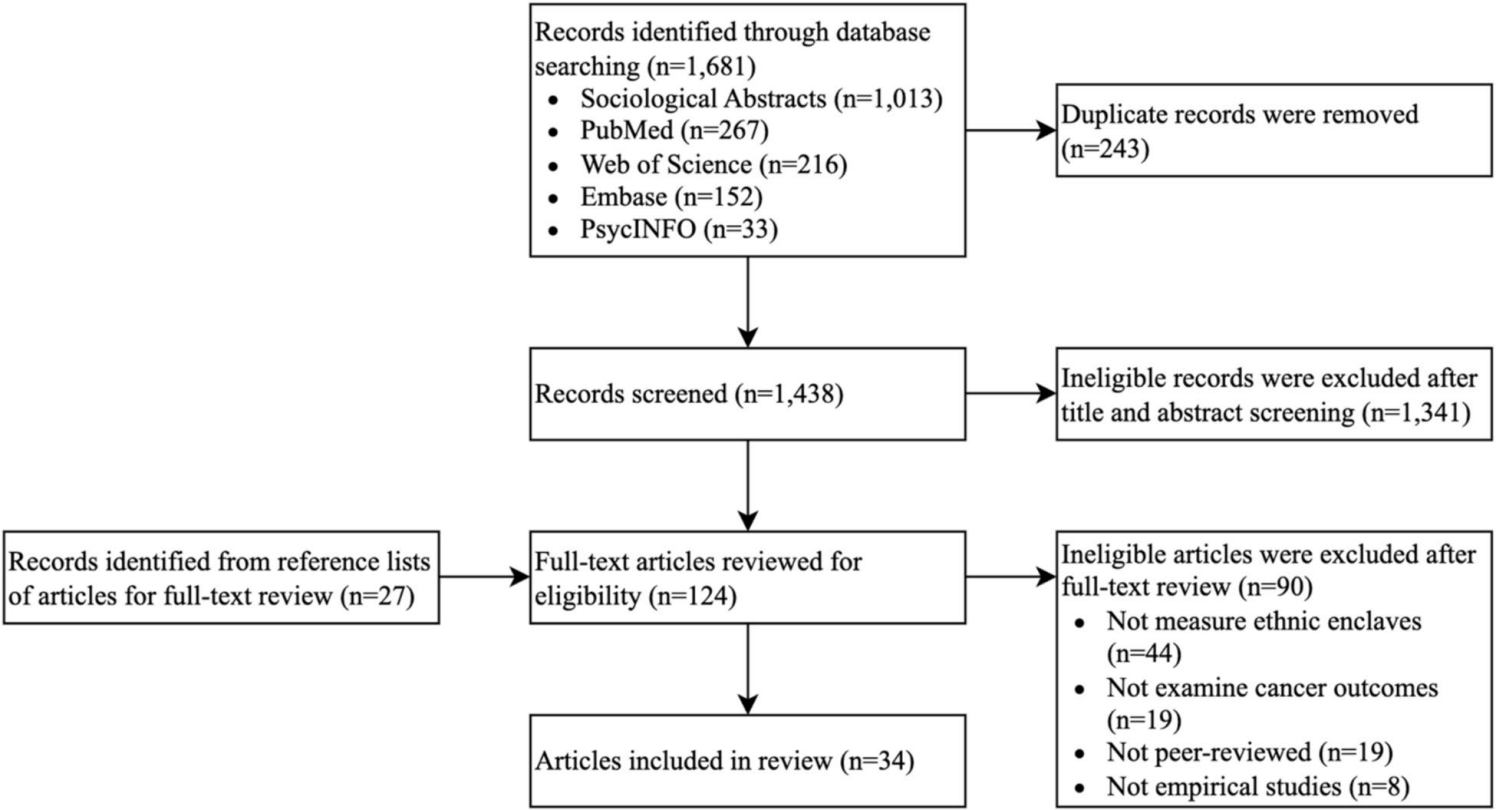
PRISMA flow diagram

After articles (*n* = 1,681) were identified through database searching and duplicates (*n* = 243) were removed, the titles and abstracts of the remaining articles (*n* = 1,438) were screened. To be included in this review, a study met the following criteria: (1) was published in peer-reviewed journals; (2) was an empirical study; (3) included sample participants in the U.S.; (4) measured sample participants’ EE residence; (5) aimed to examine the relationships between EE residence and outcomes at any point in the cancer continuum, including cancer incidence, stage at diagnosis, treatment, and survival/mortality. Ineligible articles (*n* = 1,341) that did not meet these criteria were then excluded, and the remaining articles’ (*n* = 97) full texts were assessed to determine their eligibility. To provide a more comprehensive search for relevant studies, we screened the reference lists of articles included for full-text review and identified additional 27 studies. A total of 124 articles were screened on their full texts and, finally, 34 eligible articles were included in the review. The screening process is described in Fig. 1. Sample participants, study area, EE measurement, cancer outcomes, and main findings on enclave-cancer associations, of each study were extracted for analysis (see Online Resource 1).

## Results

Studies examined the associations between EE residence and cancer outcomes among cancer patients with different races or ethnicities in different regions. Most studies (*n* = 30) [21–50] examined associations between EE residence and cancer outcomes among patients in California, one of the most racially and ethnically diverse states in the U.S. A few studies investigated the enclave-cancer associations in Texas (*n* = 5) [37, 46, 51–53], Florida (*n* = 1) [37], and New Jersey (*n* = 1) [54]. Studies mainly examined the enclave-cancer associations among Hispanics (*n* = 27) [21–23, 25–32, 35, 37, 38, 40, 41, 44–54] and Asian or Asian and Pacific Islanders (APIs) (*n* = 16) [22, 24–26, 31–34, 36, 38, 39, 42–44, 49, 50]. Only one study included Black patients [25]. Patients were identified from state-wide population-based cancer registries that provide comprehensive information about all cancer cases, except that one study [52] collected patients’ information from a hospital in Texas.

Studies also assessed the enclave-cancer associations for different cancer types and outcomes across the continuum (see Table 1). Breast (*n* = 8) [21, 33, 34, 40, 41, 43, 46, 47] and colorectal (*n* = 6) [29, 30, 39, 42, 48, 54] cancers were frequently studied. Different outcomes of liver (*n* = 3) [22, 44, 50], lung (*n* = 2) [26, 36], cervical (*n* = 2) [31, 35], gastric (*n* = 2) [23, 51], prostate cancers (*n* = 2) [28, 45], childhood leukemia (*n* = 2) [52, 53], lymphoma (*n* = 2) [24, 32], endometrial (*n* = 1) [49], thyroid (*n* = 1) [38], testicular cancers (*n* = 1) [27], melanoma (*n* = 1) [37], and childhood brain tumor (*n* = 1) [25] were also investigated. Most studies focused on the relationships between EEs and cancer incidence (*n* = 14) [21–24, 26, 27, 31, 32, 38, 40, 42–44, 50] or survival outcomes (*n* = 15) [21, 28, 30, 33, 35, 36, 39, 41, 45–49, 51, 53]. Many fewer studies investigated cancer stage at diagnosis (*n* = 4) [35, 37, 41, 54] or treatment (n = 5) [25, 29, 30, 34, 52]. The following sections discuss different methods used for determining patients’ EE residence and summarize findings on the enclave-cancer associations by cancer outcomes across the continuum.

**Table 1:** List of Studies by Ethnic Enclaves and Cancer Outcomes

Ethnic Enclaves	Incidence	Stag**e**	Treatment	Survival/Mortality
Asian or API Enclaves	Breast cancer: Morey et al. 2022 Cervical cancer: Froment et al. 2014 Colorectal cancer: Ladabaum et al. 2014 Liver cancer: Chang et al. 2010, Sangaramoorthy et al. 2022, Yang et al. 2018 Lung cancer: DeRouen et al. 2018 Lymphoma: Clarke et al. 2011, Glaser et al. 2015 Thyroid cancer: Horn-Ross et al. 2014		Brain tumor: Cooney et al. 2018 Breast cancer: Gomez et al. 2012	Breast cancer: Gomez et al. 2010 Colorectal cancer: Kcomt and Gorey 2020 Endometrial cancer: Von Behren et al. 2018 Lung cancer: Gomez et al. 2016
Hispanic Enclaves	Breast cancer: Banegas et al. 2014, Keegan et al. 2010a Cervical cancer: Froment et al. 2014 Gastric cancer: Chang et al. 2012 Liver cancer: Chang et al. 2010, Sangaramoorthy et al. 2022, Yang et al. 2018 Lung cancer: DeRouen et al. 2018 Lymphoma: Glaser et al. 2015 Testicular cancer: DeRouen et al. 2020 Thyroid cancer: Horn-Ross et al. 2014	Breast cancer: Keegan et al. 2010b Cervical cancer: Gomez et al. 2015 Colorectal cancer: Gomes et al. 2023 Melanoma: Harvey et al. 2017	Brain tumor: Cooney et al. 2018 Colorectal cancer: Escobar et al. 2019, Escobar et al. 2020 Leukemia: Muñiz et al. 2022	Breast cancer: Banegas et al. 2014, Keegan et al. 2010b, Shariff-Macro et al. 2020, Shariff-Macro et al. 2021 Cervical cancer: Gomez et al. 2015 Colorectal cancer: Escobar et al. 2020, Tao et al. 2014 Endometrial cancer: Von Behren et al. 2018 Gastric cancer: Ju et al. 2022 Leukemia: Schraw et al. 2021 Prostate cancer: DeRouen et al. 2022, Schupp et al. 2014
Black Enclaves			Brain tumor: Cooney et al. 2018	

### Methods for Defining EE Residence

All studies defined and measured EEs at the census tract or block group level; these two geographic levels have been commonly used in health disparity studies to capture neighborhood contexts and often yielded comparable results [55, 56]. Patients’ residence in EEs was determined based on their addresses at the time of cancer diagnosis. Only one study [54] accounted for patients’ residential histories prior to diagnosis and measured the length of time living in EEs. When defining EEs and assessing enclave-cancer associations, most studies treated Hispanics or Asians as one racial/ethnic group, without distinguishing subgroup differences (e.g., Chinese, Korean, Japanese, etc.). In the two studies focusing exclusively on Chinese Americans [36, 39], one measured residence in either specifically Chinese or generally Asian enclaves [39], while the other only examined patients’ residences in Asian enclaves [36].

Studies adopted three primary types of methods to operationalize and identify EEs, and the use of each method is briefly explained below. Principal component analysis (PCA) was the most used method (*n* = 27) [21–27, 31–36, 38, 40–46, 48–53], followed by the threshold-based method (*n* = 5) [29, 30, 37, 39, 54], and latent class analysis (LCA) (*n* = 2) [28, 47]. Table 2 lists the studies by the race and ethnicity of enclaves and methods of measurement.

**Table 2:** List of studies by ethnic enclaves and measurement methods

Ethnic Enclaves	Principal Component Analysis	Threshold-based Method	Latent Class Analysis
Asian or API Enclaves	Chang et al. 2010, Clarke et al. 2011, Cooney et al. 2018, DeRouen et al. 2018, Froment et al. 2014, Glaser et al. 2015, Gomez et al. 2010, Gomez et al. 2012, Gomez et al. 2016, Horn-Ross et al. 2014, Ladabaum et al. 2014, Morey et al. 2022, Sangaramoorthy et al. 2022, Von Behren et al. 2018, Yang et al. 2018	Kcomt and Gorey 2020	
Hispanic Enclaves	Banegas et al. 2014, Chang et al. 2010, Chang et al. 2012, Cooney et al. 2018, DeRouen et al. 2018, DeRouen et al. 2020, Froment et al. 2014, Glaser et al. 2015, Gomez et al. 2015, Horn-Ross et al. 2014, Ju et al. 2022, Keegan et al. 2010a, Keegan et al. 2010b, Muñiz et al. 2022, Sangaramoorthy et al. 2022, Schraw et al. 2021, Schupp et al. 2014, Shariff-Macro et al. 2020, Tao et al. 2014, Von Behren et al. 2018, Yang et al. 2018	Escobar et al. 2019, Escobar et al. 2020, Harvey et al. 2017, Gomes et al. 2023	DeRouen et al. 2022, Shariff-Macro et al. 2021
Black Enclaves	Cooney et al. 2018		

Studies used PCA to construct EE indices by combining multiple sociodemographic variables to identify the extent to which each geographic area (i.e. census tract or block group) was ethnically distinct [57]. Such variables included racial or ethnic composition, immigrant population, English proficiency, and linguistic isolation, from Census and/or American Community Surveys (ACS). Studies usually categorized census tracts or block groups into two to five groups based on the state-wide distribution of EE indices. Areas with higher index values were more ethnically distinct (i.e. EEs), whereas those with lower index values were less ethnically distinct (i.e. non-enclaves). As EE status and neighborhood SES were often correlated and might exert joint effects on cancer risks, some studies (*n* = 17) [22–24, 26, 27, 31, 32, 34, 35, 38, 40–44, 46, 49] combined EE status and neighborhood SES levels to classify areas into different types (e.g., low SES enclaves, low SES non-enclaves, high SES enclaves, and high SES non-enclaves).

Another group of studies used thresholds of racial or ethnic composition to measure enclaves. Predetermined threshold values of the percentage of a specific racial or ethnic group were used to classify census tracts into enclaves and non-enclaves. In these studies, Hispanic, Asian, and Chinese enclaves in California were defined as census tracts with 40%, 30%, and 10% or more Hispanic, Asian, and Chinese population, respectively [29, 30, 39]. In addition to considering the racial or ethnic composition in each census tract individually, one study examining Hispanic enclaves in New Jersey also accounted for the geographic clustering pattern of census tracts that formed EEs. The study defined Hispanic enclaves as census tracts with 50% or more Hispanic populations and their adjacent census tracts with 20% or more Hispanic populations [54].

While most studies using thresholds focus solely on racial or ethnic population composition, one study operationalized Hispanic enclaves using thresholds for three different variables: percentage of Hispanic population, percentage of Hispanics who were foreign-born, and percentage of Hispanics with limited English proficiency [37]. The study then assessed the associations between each of the three enclave variables and the cancer outcome respectively. Across the three states (i.e., California, Florida, and Texas) where the study [37] was conducted, the percentage of Hispanic population and the percentage of Hispanics with limited English proficiency were strongly correlated across census tracts. In California and Florida, these two variables were also correlated with the percentage of foreign-born Hispanics. However, this was not the case in Texas, where Hispanic-predominant tracts had larger proportions of US-born Hispanics. These variations highlight regional variations in population characteristics related to enclave measurement.

Two studies used results from latent class analysis (LCA) to identify EEs. Unlike the PCA method that focused on a set of demographic variables, these two studies classified census tracts into nine different types based on a wide range of social and built environment characteristics, such as, demographics, SES, housing, land use, traffic, and food environment [28, 47]. For example, Hispanic enclaves were identified from the LCA as featuring high concentrations of Hispanic population and lower-middle SES.

### Associations between EE and its Impact on Cancer

This section summarizes main findings on the enclave-cancer relationships across the cancer continuum (see Table 3). As might be expected with a group of studies using divergent methods and looking at diverse ethnic groups, cancer types, and cancer outcomes, results are mixed for protective, null, and risk findings.

**Table 3:** Associations between ethnic enclave residence and cancer outcomes

**Incidence**			
**Ethnic Enclaves**	**Protective**	**Null**	**Risk**
Asian or API Enclaves	Colorectal cancer: Ladabaum et al. 2014 Lymphoma: Clarke et al. 2011 (F), Glaser et al. 2015 (F)	Liver cancer: Chang et al. 2010 (F), Sangaramoorthy et al. 2022 (F) Lung cancer: DeRouen et al. 2018 Lymphoma: Clarke et al. 2011 (M), Glaster et al. 2015 (M)	Breast cancer: Morey et al. 2022 (high SES areas) Cervical cancer: Froment et al. 2014 Liver cancer: Chang et al. 2010 (M), Sangaramoorthy et al. 2022 (M), Yang et al. 2018 Thyroid cancer: Horn-Ross et al. 2014
Hispanic Enclaves	Breast cancer: Keegan et al. 2010a Lymphoma: Glaser et al. 2015 (F) Lung cancer: DeRouen et al. 2018 Testicular cancer: DeRouen et al. 2020	Gastric cancer: Chang et al. 2012 (cardia) Liver cancer: Chang et al. 2010 (M), Sangaramoorthy et al. 2022 (M) Lymphoma: Glaser et al. 2015 (M)	Breast cancer: Banegas et al. 2014 (HR +/HER2 + vs. HR +/HER-) Cervical cancer: Froment et al. 2014 Gastric cancer: Chang et al. 2012 (non-cardia, intestinal) Liver cancer: Chang et al. 2010 (F), Sangaramoorthy et al. 2022 (F), Yang et al. 2018 Thyroid cancer: Horn-Ross et al. 2014
**Stage at Diagnosis**			
**Ethnic Enclaves**	**Protective**	**Null**	**Risk**
Hispanic Enclaves	Colon cancer: Gomes et al. 2023	Cervical cancer: Gomez et al. 2015 (high SES areas)	Breast cancer: Keegan et al. 2010b (high SES areas) Melanoma: Harvey et al. 2017
**Treatment**			
**Ethnic Enclaves**	**Protective**	**Null**	**Risk**
Asian or API Enclaves		Brain tumor: Cooney et al. 2018 Breast cancer: Gomez et al. 2012 (high SES areas)	
Hispanic Enclaves	Colon cancer: Escobar et al. 2019 (M), Escobar et al. 2020 (M)	Brain tumor: Cooney et al. 2018 Colon cancer: Escobar et al. 2019 (F), Escobar et al. 2020 (F)	Leukemia: Muniz et al. 2022
Black Enclaves		Brain tumor: Cooney et al. 2018	
**Survival/Mortality**			
**Ethnic Enclaves**	**Protective**	**Null**	**Risk**
Asian or API Enclaves	Colon cancer: Kcomt and Gorey 2020 (F)	Breast cancer: Gomez et al. 2010 Colon cancer: Kcomt and Gorey 2020 (M) Endometrial cancer: Von Behren et al. 2018 Lung cancer: Gomez et al. 2016	
Hispanic Enclaves	Breast cancer: Shariff-Macro et al. 2020 Colon cancer: Escobar et al. 2020 (M)	Breast cancer: Banegas et al. 2014 Cervical cancer: Gomez et al. 2015 Colorectal cancer: Escobar et al. 2020 (F, colon), Tao et al. 2014 Endometrial cancer: Von Behren et al. 2018 Gastric cancer: Ju et al. 2022	Breast cancer: Shariff-Macro et al. 2021; Keegan et al. 2010b (low SES enclaves vs high SES non-enclaves) Leukemia: Schraw et al. 2021 Prostate cancer: DeRouen et al. 2022, Schupp et al. 2014

Notes: *F* female, *M* male, *SES* socioeconomic status, *HR* hormone receptor, *HER* human growth factor receptor

#### EE Residence and Cancer Incidence

Studies (*n* = 14) [21–24, 26, 27, 31, 32, 38, 40, 42–44, 50] that examined the associations between EE residence and cancer incidence all used PCA to identify EEs and focused on patients in California. Despite utilizing the same enclave measurement method, the results were mixed. EEs generally had lower incidence rates of colorectal cancer [42], testicular cancer [28], lung cancer [26], and lymphoma [24, 32] than non-enclaves did, whereas the opposite pattern was noted for cervical [31], thyroid [38], liver [22, 44, 50], and non-cardia or intestinal gastric cancers [23].

Looking yet more deeply at the results, the strengths of associations differed by sex, race or ethnicity, and cancer subtype. For instance, Hispanic enclave residents had lower overall risks of developing breast cancer but elevated risks for a more aggressive subtype (i.e., hormone receptor positive (HR+) and human growth factor receptor 2 positive (HER2+) type relative to HR+/HER2-type) [21, 40]. Asian females living in high SES enclaves also had higher risks of developing breast cancer than those in high SES areas outside of enclaves [43]. These different patterns may be related to variations in SES, immigration histories, and cultures between Hispanic and Asian enclaves [43]. In addition, the enclave-incidence associations also varied by sex and race and ethnicity for lymphoma, lung cancer, and liver cancer. This may reflect varying influences of EEs on different cancer risk factors, such as reproductive experiences, smoking behaviors, obesity rates, and HBV vaccination rates, among various populations [22, 24, 26, 32, 44].

#### EE Residence and Cancer Stage at Diagnosis

Four studies, all focused on Hispanic enclaves, examined cancer stage at diagnosis, and the enclave association with stage of diagnosis differed by cancer type [35, 37, 41, 54]. Two studies, using a threshold-based method to identify Hispanic enclaves, found that Hispanic enclaves were associated with early diagnosis of colon cancer in New Jersey [54] but later diagnosis of melanoma in California, Texas, and Florida [37]. The opposite patterns may be related to improved colorectal cancer screening rates inside enclaves, benefiting from culturally competent health resources, but fewer skin examinations [37, 54]. The regional variations may also reflect the varying Hispanic enclave features and health policies across states [37].

Two studies conducted in California identified Hispanic enclaves using the PCA method; they examined the joint effects of EE and neighborhood SES on cervical and breast cancers stage at diagnosis, respectively [35, 41]. In high SES areas, Hispanic enclave residence was associated with a later diagnosis of breast cancer but not cervical cancer [35, 41]. Older populations, who are more likely to be affected by breast cancer than cervical cancer, and who are living in EEs may be reluctant to receive cancer screenings due to cultural values like fatalism and cancer fear [14].

#### EE Residence and Cancer Treatment

Five studies [25, 29, 30, 34, 52] assessed the associations between EE residence and cancer treatment; findings also varied by cancer type and sex. Hispanic enclave residence was associated with better receipt of chemotherapy for colon cancer among males but not females, potentially due to the stronger family support that males inside enclaves received from spouses [29, 30]. However, living in Hispanic enclaves was associated with worse treatment outcomes among children with acute lymphoblastic leukemia (ALL) [52]. The treatment pattern did not differ by enclave status among Hispanic, API, or Black children with high-grade glioma and/or medulloblastoma [25]. A study examined the joint effects of Asian enclave status and neighborhood SES on breast cancer treatment patterns (i.e., receiving mastectomy or breast-conserving surgery without radiation compared to breast-conserving surgery with radiation); no significant differences were noted between high SES enclaves and non-enclaves [34].

#### EE Residence and Cancer Survival/Mortality

For both Asians and Hispanics, the enclave-survival associations mainly varied by cancer type. Living in Asian enclaves was not significantly associated with survival outcomes among patients diagnosed with breast, endometrial, or lung cancers [33, 36, 49]. The protective effect of Asian enclave residence on colon cancer survival outcomes was only observed among Chinese females, but not males [39].

The relationships between Hispanic enclave residence and colon cancer [30], cervical cancer [35], or ALL [53] survival outcomes were similar to those patterns noted in studies on cancer stage at diagnosis or treatment, indicating the important roles of early diagnosis and quality treatment on cancer survival outcomes. Endometrial, gastric, and colorectal cancer survival outcomes did not differ by Hispanic enclave residence status [48, 49, 51]. However, living in Hispanic enclaves was associated with worse prostate cancer survival possibly due to unhealthy food environment in these enclaves [28, 45]. The overall breast cancer survival outcomes were better in Hispanic enclaves, while the subtype-specific mortality did not differ by enclave status [21, 46]. When combining Hispanic enclave status and neighborhood SES, studies found that patients living in low SES enclaves had worse breast cancer survival than those in high SES non-enclaves [41, 46, 47].

## Discussion

The heavily mixed findings in the literature to date present some signals of potentially important associations between EEs and cancer outcomes. However, much more research is needed to separate actionable signals from noise.

### EE Conceptualization and Operationalization in Cancer Studies

EEs have been conceptualized in different ways in previous literature. Sociologists have traditionally defined EEs based on ethnic economy (e.g., ethnic-owned businesses) and the concentration of immigrants (e.g., “immigrant enclaves”) [8, 58–60]. Many EEs arose from immigrant settlement and developed as immigrants started ethnic businesses, gained social capital, and established cultural ties [8, 61]. Such immigrant enclaves often emerged as a result of social exclusion and discrimination, locating in inner cities and having low SES [7, 10, 62]. Later, some immigrant enclave residents and their later generations may move to non-enclave neighborhoods after improving SES, in a process known as spatial assimilation [63]. Other EEs result from residential preferences as racial and ethnic minorities choose to live in ethnically concentrated neighborhoods even when moving to non-enclave neighborhoods is possible due to achieving higher SES [7, 62]. Many of these EEs formed in suburban areas, and are known as “ethnoburbs” [7, 11]. Such ethnoburbs attract both new immigrants with higher SES and some later-generation descendants of immigrants [10, 11].

Despite such conceptual richness in the broader literature on EEs, the reviewed studies often lacked in-depth discussions on how they approached the concept of EE and how their operationalization of EEs mirrored the EE definition. Most studies identified EEs relying on area-level demographic characteristics, such as racial or ethnic population compositions, immigrant concentrations, and linguistic isolation, overlooking the nuances among different forms of EEs. Moreover, the reviewed studies adopted different methods (i.e., PCA, population thresholds, and LCA) to determine EEs. While the findings on enclave-cancer associations among the 34 studies did not systematically vary by the method for measuring EEs, given the small number of studies, it is not possible to draw reliable conclusions on how different EE measures may affect findings of the enclave-cancer associations.

To better compare enclave-cancer relationships across varying contexts and to further the understanding of how EEs affect various cancer outcomes, it is necessary for future studies to clarify the EE definition and construct an EE measure that reflect its demographic, cultural, geographic characteristics and complexities. We propose an EE definition could be applied to cancer studies: areas where spatial clusters of concentrated population from a certain racial and ethnic group and ethnic-specific businesses and/or organizations are co-located. While a few reviewed studies acknowledged that EEs often featured with the prevalence of ethnic-specific businesses and social institutions [44, 46, 51, 53], the presence of these cultural landscapes were not included in their EE measurements. Considering cultural landscapes of EEs are critical for cancer studies because they may exert influences on residents’ cancer risks through mechanisms that may not be captured by population-based enclave measures. They may provide employment opportunities for new immigrants and residents with limited English proficiency as well as ethnic-specific resources, such as traditional food options and culturally competent health providers; these resources may then influence residents’ cancer risks through insurance coverages, diets, and healthcare access and utilization [8, 13, 64]. Additionally, recent studies have shown that areas with high concentrations of ethnic-specific businesses and organizations do not necessarily have the highest racial and ethnic minority and/or immigrant population concentrations [64]. Thus, it is necessary for future cancer studies to account for distributions of both ethnic populations and businesses or organizations when delineating EE boundaries.

Following this broad EE definition, we acknowledge that EEs have different forms, varying in SES, immigrant concentration, and composition of disaggregated racial or ethnic subgroups. These different EEs may have distinct social and built environment, leading to varying associations with health outcomes [10, 37]. Distinguishing heterogeneity across EEs may help disentangle some mixed findings on the complex enclave-cancer associations. Some reviewed studies separated low SES EEs and high SES EEs when examining their associations with cancer outcomes [23, 24, 26–28, 31–35, 38, 40–44, 46, 47, 49]. Variations in immigrant concentrations and racial or ethnic subgroup compositions across EEs were rarely addressed in cancer studies. One study used three different EE measures and investigated their associations with melanoma diagnosis separately; the study found that Hispanic enclaves defined by Hispanic immigrant concentration and enclaves defined by Hispanic population concentration (regardless of nativity) had different associations with melanoma late-stage diagnosis, particularly in states where many Hispanic population enclaves had high percentages of US-born Hispanics, which indicated the important roles of area-level immigrant concentrations in melanoma diagnosis [37]. While some reviewed studies considered the percentage of immigrant population when constructing the enclave index using PCA, these studies did not distinguish EEs with different immigrant concentrations. An EE typology proposed in previous literature could be applied to cancer studies to capture nuances across EEs [10].

Additionally, current studies often follow broad racial and ethnic categories, such as Hispanics or Asians, when identifying EEs. Only one study distinguished Chinese enclaves and Asian enclaves and found stronger protective effects of Chinese enclaves than Asian enclaves on colon cancer survival among Chinese patients [39]. Hispanics or Asians in the U.S. consist of populations with various sociodemographic characteristics, immigration histories, cultural backgrounds, and cancer risks, forming different types of EEs and resulting in unique health impacts [10, 26, 43]. Therefore, the experiences of living in Asian or Hispanic enclaves dominated by different subgroups may not be identical. It is necessary to distinguish EEs with differing racial/ethnic subgroup compositions and immigrant concentrations, as well as to assess the influences of EEs by patient race and ethnicity subgroups.

The operationalization of EEs in the reviewed studies are often aspatial. Most studies determined EE status by focusing on population characteristics within each individual census tract or block group without investigating demographic compositions in adjacent areas. Some sociologists and human geographers emphasized that EEs often span areas larger than a single census tract, which helps build social ties among co-ethnic populations and supports the growth of ethnic businesses and organizations [7, 10]. However, only one reviewed study considered neighboring census tracts when identifying EEs [54]. Future studies may better delineate EE boundaries by accounting for the geographic connectivity and spatial clustering of ethnic populations and ethnic businesses or organizations as EEs were formed. Some spatial methods, such as local indicators of spatial association (LISA), that evaluate local clustering patterns can be useful to identify EEs [7, 10, 65].

Furthermore, using census tract or block group as the geographic unit for EEs may subject to the modifiable areal unit problem (MAUP) or the uncertain geographic context problem (UGCoP). The MAUP refers to bias related to aggregation of values into different geographic scales or using different boundaries [66], while the UGCoP may arise when the geographic boundaries of analysis may not reflect the true geographic context [67]. Census tract boundaries may not align with residents’ perception of EE boundaries and different cancer patterns may be observed when aggregating into different geographic scales [68, 69]. These problems may further complicate the interpretations of observed enclave-cancer relationships. Future studies may benefit from constructing and comparing EE measures using different geographic units as well as applying qualitative methods that involve community members in delineating their perceived EE boundaries.

Accurately and comprehensively operationalizing EEs is particularly challenging, and previous studies have been constrained by limited availability of data, such as lack of fine-scale data on disaggregated racial and ethnic subgroup populations. Future studies may take advantage of emerging spatial data, including mobility data, points of interest data, and street view images, to address some limitations in current studies and refine EE measurements. For instance, the street view images capture the built environment of neighborhoods at the street level and machine learning techniques allow researchers to effectively extract various neighborhood characteristics from images, which could be applied to characterizing cultural landscapes within EEs [70–72]. The mobility data and points of interest data are useful to describe EE residents’ travel behaviors to ethnic-specific businesses and organizations, providing insights on access to and utilizations of ethnic resources [73].

### Relationships between EE Residence and Cancer Risks

While there has been growing interest in exploring the complex relationships between EE residence and cancer outcomes, empirical examinations of the associations are still limited and current findings on the enclave-cancer relationships are inconsistent. Current studies showed that the enclave-cancer associations mainly differed by cancer type and outcomes across the continuum. Living in EEs was generally associated with improved colon cancer outcomes, including lower incidence rates, early diagnosis, better treatment, and improved survival outcomes, particularly among Hispanic males and Asian females. Findings on EE residence and breast cancer outcomes were mixed; the associations varied by tumor subtype, patients’ race or ethnicity, and neighborhood SES. Studies on other cancers were too few to draw meaningful conclusions. Furthermore, current studies frequently focused on cancer incidence and survival outcomes, but more studies are needed to examine EEs’ influences on stage at diagnosis and treatment. Late-stage diagnosis of some cancers (e.g., colorectal cancer, cervical cancer) may be preventable through regular screening. Timely diagnosis and quality treatment may have significant impacts on survival outcomes. Thus, understanding the role of EE residence on cancer diagnosis and treatment have important implications for designing effective cancer control programs to reduce cancer burden. Lastly, existing research on enclave-cancer relationships mainly focused on Hispanic and Asian populations in California. However, these observed associations may not be generalizable to other racial and ethnic groups or states, due to diverse population characteristics, immigrant histories, and cultural backgrounds. Therefore, there are many opportunities for future studies to explore the associations among various populations in different regions who are diagnosed with or at risk of developing different cancers.

Neighborhood environment may interact with other individual- and/or area-level social determinants of health and exert influences on cancer outcomes[5]. More studies are needed to explore potential moderators and mediators between EEs and cancer outcomes. Current studies showed that the relationships between EE residence and cancer outcomes may vary by other demographic, tumor, and geographic characteristics (i.e. moderators), including sex, nativity, marital status, age, tumor subtype, and neighborhood SES. Sex and neighborhood SES were often assessed in existing studies. The associations between EE residence and Hodgkin and non-Hodgkin lymphoma incidence, liver cancer incidence, and colon cancer treatment and survival were found to differ by sex [22, 24, 29, 30, 32, 39, 44]. The protective effects of EEs on lymphoma, lung, or testicular cancer incidence, breast or colon cancer survival were commonly reported among patients in low SES areas [26, 27, 30, 32, 46], highlighting that EEs may provide critical support for these vulnerable populations and buffer the potentially negative effects of living in deprived areas. Fewer studies explored the enclave-cancer associations stratified by nativity, age, marital status, and tumor subtype. Further examinations of these moderators may help explain some of the mixed findings on enclave-cancer associations noted among different groups of cancer patients, and identify populations and areas with higher risks needed targeted cancer control resources.

Studies discussed possible mechanisms through which EEs influence different cancer outcomes. These mediators include access to culturally competent healthcare resources that affect cancer screening uptake as well as lifestyles (e.g., smoking, pregnancy history) relevant to cancer etiology [22, 24, 26, 32, 44, 54]. However, hypotheses on these mediation effects are often not empirically tested in the reviewed studies. Only one study examined mediators, including patients’ SES, mammogram utilization, health behaviors, and neighborhood environment, for the relationship between Asian enclave residence and breast cancer incidence, and did not find significant results [43]. Future studies might usefully examine additional mediators, such as social networks, experiences of discrimination and stress, food environment and diet, physical activities, and health literacy and beliefs [6, 12, 13, 15, 43]. Mixed methods, including mediation analysis, multi-level modeling, interviews of cancer patients living within and outside of EEs, and social network analysis, would be helpful to understand cancer-related facilitators and barriers that EE residents face and to establish the causal link between EEs and cancer outcomes.

Furthermore, cancer is a chronic disease and EE residence may have cumulative influences on the development and progression of the disease. With the increasing availability of residential history data linked to cancer registry data, there is growing attention to exploring patients’ long-term exposure to environmental risks [74]. However, only one reviewed study accounted for patients’ long-term residence within EEs before diagnosis [54] while all other studies examined EE residence at the time of diagnosis and its relationships to cancer outcomes. Future studies may benefit from using longitudinal data to track patients’ residential choices prior to diagnosis and throughout their cancer journey to better understand EEs’ impacts on cancer outcomes in the long run.

## Conclusion

While this systematic review conducted an extensive search of relevant empirical studies in multiple databases, it may have missed some studies that focused on similar residential patterns, such as high ethnic density neighborhoods or racial segregation, and did not self-identify as applying the concept of EE. A previous review of cancer studies broadly summarized findings on the impacts of neighborhood ethnic density and racial segregation [17]. In contrast, this review specifically focuses on the conceptualization and operationalization of EE, as a neighborhood-level social determinants of health that may influence residents’ cancer risks. Current findings on enclave-cancer associations were inconsistent across studies. Tumor, demographic, and geographic characteristics, such as cancer type, tumor subtype, race and ethnicity, sex, and neighborhood SES may modify the enclave-cancer associations. Changes in population compositions, neighborhood contexts, health programs, and cancer diagnosis or treatment options over space and time further complicated the findings.

Given the complex enclave-cancer relationships and the limited number of studies published, the EE effects on cancer remain an under-researched area and more studies are needed to address methodological challenges and fill knowledge gaps. Due to the complexities of cancer that represents multitudes of different types of diseases, more studies are needed to draw conclusions on enclave-cancer associations for different cancer types and outcomes. Specifically, future studies may examine cancer types with mixed findings (e.g., breast cancer) or limited evidence (e.g., lung cancer, cervical cancer) in existing literature, and different outcomes, especially cancer diagnosis and treatment. Research also needs to clearly define EEs and carefully operationalize it, possibly achieving convergence in the measurement of EEs. The varying EE measures adopted by the reviewed studies make it difficult to directly compare the effect sizes of enclave-cancer associations. Applying comparable enclave measurement in future cancer studies will help establish a solid foundation for future reviews and meta-analyses. To advance the understanding of EEs’ long-term impacts on cancer, future studies may take advantage of longitudinal data and mixed methods to explore both moderators and mediators between EEs and cancer outcomes.

Progress in research on EEs and cancer has public health implications for policymakers and health professionals who are interested in learning about population and community characteristics for improving cancer control efforts. Understanding both risk and protective factors of EE residence on cancer outcomes will likely provide important information to design more effective cancer prevention and control programs that target different population groups’ health needs and leverage social structures and neighborhood resources.

## Supplementary Information

Below is the link to the electronic supplementary material.

Supplementary file1 (XLSX 19 KB)

## Data Availability

Review materials are available upon request.
